# Supplementary material for: Predicting visual function by interpreting a neuronal wiring diagram
Source: Nature. 2024 Oct 2;634(8032):113–23. doi: 10.1038/s41586-024-07953-5 (PMC11446822; doi:10.1038/s41586-024-07953-5)
Supplement: Supplementary file 6 — CRF and ERF predictions for individual TmY4 and TmY9 cells. Analogous to Supplementary Data 3, but for TmY target types. Shown are the top four monosynaptic pathways, the strongest pathway passing through each of the top ten intermediary types (ranking from Extended Data Fig. 7), and the trisynaptic pathway Tm1–TmY–Dm3–TmY (see the section entitled Prediction of spatial normalization). [file 41586_2024_7953_MOESM6_ESM.zip › DataS4/TmY9q/720575940616513695.pdf]

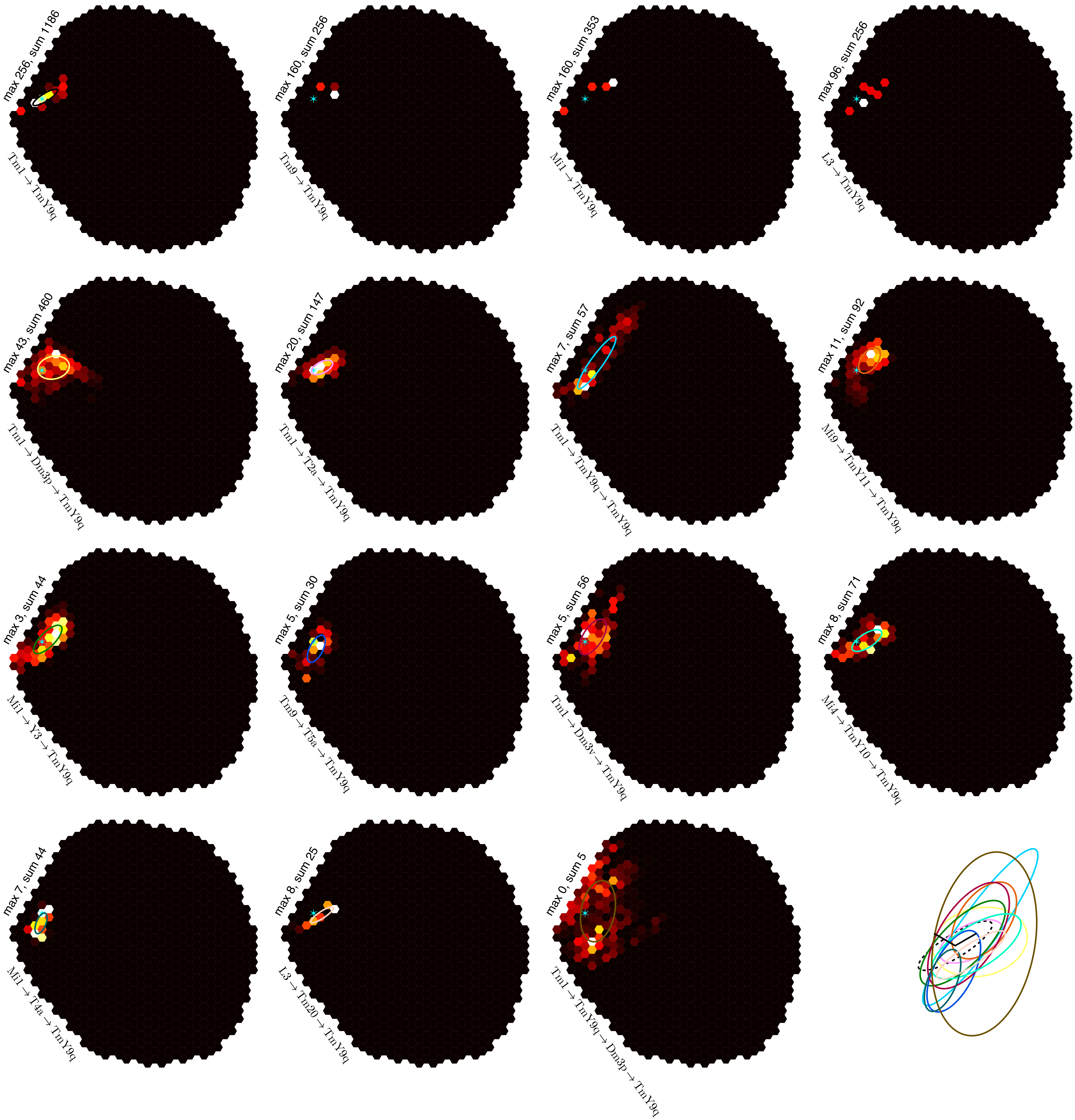
max 256, sum 1186
Tm1 → TmY9q
max 160, sum 256
Tm9 → TmY9q
max 160, sum 353
Mi1 → TmY9q
max 96, sum 256
L3 → TmY9q
max 43, sum 460
Tm1 → Dm3p → TmY9q
max 20, sum 147
Tm1 → T2a → TmY9q
max 7, sum 57
Tm1 → TmY9q → TmY9q
max 11, sum 92
Mi9 → TmY11 → TmY9q
max 3, sum 44
Mi1 → Y3 → TmY9q
max 5, sum 30
Tm9 → T5a → TmY9q
max 5, sum 56
Tm1 → Dm3v → TmY9q
max 8, sum 71
Mi4 → TmY10 → TmY9q
max 7, sum 44
Mi1 → T4a → TmY9q
max 8, sum 25
L3 → Tm20 → TmY9q
max 0, sum 5
Tm1 → TmY9q → Dm3p → TmY9q
